# Mechanisms of hypervirulent *Clostridium difficile* ribotype 027 displacement of endemic strains: an epidemiological model

**DOI:** 10.1038/srep12666

**Published:** 2015-07-28

**Authors:** Laith Yakob, Thomas V. Riley, David L. Paterson, John Marquess, Ricardo J. Soares Magalhaes, Luis Furuya-Kanamori, Archie C.A. Clements

**Affiliations:** 1 London School of Hygiene and Tropical Medicine, Department of Disease Control, London, Keppel Street WC1E 7HT; 2 Department of Microbiology, Queen Elizabeth II Medical Centre, The University of Western Australia, Nedlands, WA, Australia 6009; 3 The University of Queensland, UQ Centre for Clinical Research, Herston, Queensland, Australia 4029; 4 Communicable Diseases Unit, Queensland Department of Health, Herston, QLD, Australia 4006; 5 School of Veterinary Science, University of Queensland, Gatton, Australia 4343; 6 Children’s Health and the Environment Program, Queensland Children’s Medical Research Institute, The University of Queensland, Herston, Queensland, Australia; 7 Research School of Population Health, Australian National University, Canberra, ACT, Australia

## Abstract

Following rapid, global clonal dominance of hypervirulent ribotypes, *Clostridium difficile* now constitutes the primary infectious cause of nosocomial diarrhoea. Evidence indicates at least three possible mechanisms of hypervirulence that facilitates the successful invasion of these atypical strains: 1) increased infectiousness relative to endemic strains; 2) increased symptomatic disease rate relative to endemic strains; and 3) an ability to outcompete endemic strains in the host’s gut. Stochastic simulations of an infection transmission model demonstrate clear differences between the invasion potentials of *C. difficile* strains utilising the alternative hypervirulence mechanisms, and provide new evidence that favours certain mechanisms (1 and 2) more than others (3). Additionally, simulations illustrate that direct competition between strains (inside the host’s gut) is not a prerequisite for the sudden switching that has been observed in prevailing ribotypes; previously dominant *C. difficile* strains can be excluded by hypervirulent ribotypes through indirect (exploitative) competition.

*Clostridium difficile* is a globally significant enteric pathogen with rapid emergence in the Americas, Asia, Oceania and Europe^1^. It is reported to be the leading cause of infectious diarrhoea in healthcare facilities of developed nations^2^, and the burden of disease caused by this pathogen is receiving increasing recognition. Disease severity ranges from asymptomatic infection to potentially fatal conditions including toxic megacolon, bowel perforation and sepsis.

In 2005, when performing a Europe-wide survey of 38 hospitals in 14 countries, the European Study Group of *C. difficile* found a novel ribotype (BI/NAP1/027) in Ireland, the Netherlands and Belgium^3^. Within 3 years this PCR ribotype had spread to at least 16 European countries^4^ and was rapidly becoming one of the more prominent strains in North America^5^.

Ribotype 027 was the causative agent of the largest *C. difficile* epidemic recorded to date, in which over 2000 fatalities occurred in Quebec, Canada during 2005. Early reports of this outbreak described higher-than-expected rates of morbidity and mortality associated with ribotype 027, giving rise to the term “hypervirulent” to distinguish this strain (and, subsequently, other strains such as ribotype 078) from “typical” endemic strains. Clear disambiguation between hypervirulent and typical strains is currently precluded by incomplete understanding of what causes some strains to generate outbreaks with substantial morbidity. We use ribotype 027 to demonstrate the invasion dynamics of hypervirulent strains because it was the causative agent of the largest recorded outbreak of *C. difficile* and because the considerable literature pertaining to this particular strain facilitates more accurate model parameterisation.

Over the past decade, research has been conducted to understand hypervirulence in *C. difficile* with no consensus reached on precise causative mechanisms. Here, three plausible explanations for the increased virulence associated with some newly emergent strains of the pathogen (including ribotype 027) are summarised.

**Hypervirulent strains are more infectious than endemic strains**

Pathogen transmission is via the fecal-oral route with new infections arising from the consumption of bacterial spores. *C. difficile* spores are highly desiccation resistant and can persist on hard surfaces for as long as 5 months^6^,^7^. *In vitro* studies conducted by Merrigan and colleagues^8^ examined the accumulation of spores over the bacterial growth cycle and demonstrated that hypervirulent strains sporulated earlier and accumulated significantly more spores per total volume of culture than non-hypervirulent strains. This increased rate of sporulation may explain, at least in part, the observation of unusually high relapse rates associated with hypervirulent strains (in the order of 4-fold according to Marsh *et al.*^9^) because patients are more likely to contaminate their local environment and subsequently re-infect themselves. However, due to recent evidence to the contrary, the notion of enhanced sporulation in hypervirulent strains remains contentious^10^.

**Hypervirulent strains result in a higher rate of symptomatic disease**

Following ingestion of the dormant bacterial stage, the *C. difficile* spore germinates on exposure to bile salts in combination with L-glycine^11^,^12^. Vegetative growth of the bacterium occurs during colonization of the host’s gut. While colonization is a prerequisite of disease, most colonized individuals remain asymptomatic. Clinical manifestations of *C. difficile* disease are mediated through the production of toxins that are cytotoxic to epithelial cells of the large intestine, causing extensive colonic inflammation and epithelial tissue damage to the host^13^. Studies conducted by Pépin and colleagues^14^ and Hubert *et al.*^15^ both describe a doubling in the rate of complicated cases (severe disease) during the rise of ribotype 027 in Canada. Higher rates in symptomatic disease associated with hypervirulent strains have been postulated to result from increased toxin production^16^,^17^ or possibly through heightened activity in variant forms of clostridial toxins^18^. It is important to note that there is also contention surrounding the notion of more disease (relative to asymptomatic carriage) and worse disease outcomes from hypervirulent infections^19^,^20^.

**Hypervirulent strains can outcompete endemic strains in the host’s gut**

Recently, Robinson and colleagues (2014) tested the hypothesis that vegetative cells of hypervirulent *C. difficile* strains could outcompete endemic strains for niche space. Four ribotype 027 clinical isolates and clinical isolates of four other strains (001, 002, 014 and 053) were pairwise tested in human fecal bioreactors and in a humanized microbiota mouse model. Ribotype 027 strains outcompeted endemic strains both *in vitro* and *in vivo* and the authors postulated that this competitive advantage is key to the overrepresentation of 027 in recent outbreaks^21^.

To offer unique perspective to the critical epidemiological question of which mechanism underlies the rapid global spread (and for many regions, the subsequent clonal dominance) of ribotype 027, we analysed the simulated invasion of hypervirulent *C. difficile* following its introduction into a human community.

## Methods

A Direct Gillespie algorithm was scripted in Matlab® software version 7.12 to simulate the epidemiological state transitions involved in *C. difficile* infection with endemic and hypervirulent strains. The simulated introduction of a hypervirulent strain into a community already harbouring endemic *C. difficile* used transmission parameters that were informed by the clinical literature (see Table 1). Following the numerical recipe outlined by Keeling and Rohani (2007), an exact stochastic analogue of the following set of ordinary differential equations was constructed:
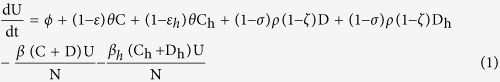

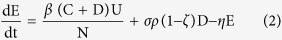

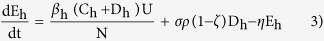

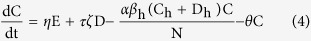

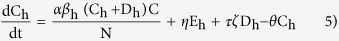

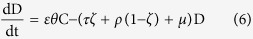

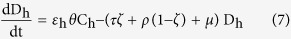


**Figure 1:**
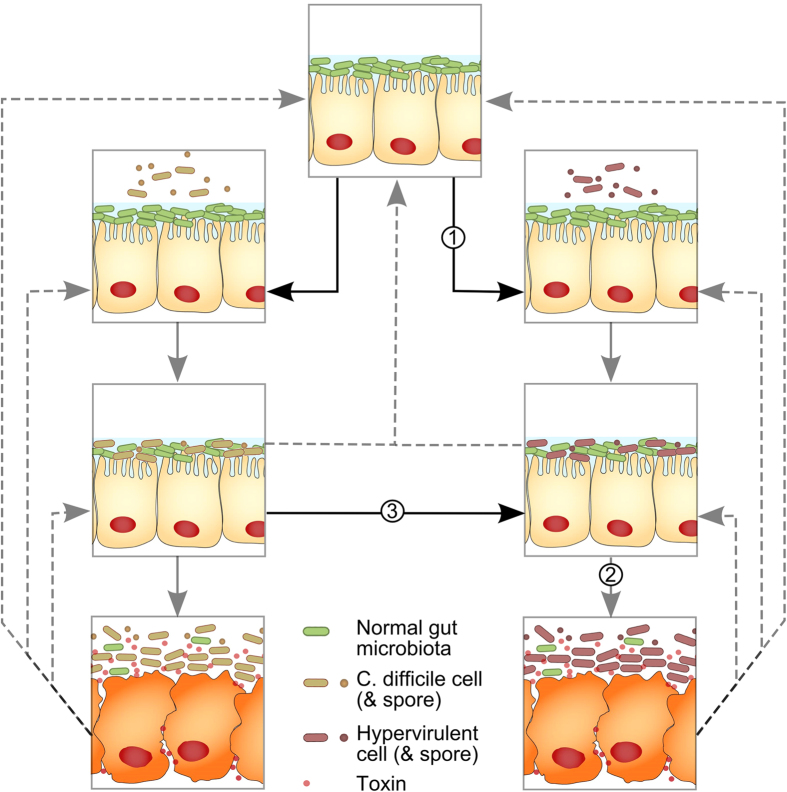
The *Clostridium difficile* epidemiological state transitions simulated by the stochastic model. The left column details colonisation with ‘typical’ endemic strains and the right column details colonisation with hypervirulent strains. From reviewing the literature, three possible areas were identified that differentiated transitions in hypervirulent strains from endemic strains: 1) Hypervirulent strains are more infectious (modelled by increasing the transmission coefficient); 2) Hypervirulent strains give rise to a higher symptomatic rate; and 3) Hypervirulent strains can outcompete endemic strains in the host’s gut.

The equations describe the rates of change between the different epidemiological categories as summarized in Fig. 1. These categories consist of people who are unexposed to *C. difficile* and who are susceptible to colonization (U); exposed to endemic strains (E) or to hypervirulent strains (E_h_); colonized with endemic strains (C) or with hypervirulent strains (C_h_); and suffering symptomatic disease from endemic strains (D) or hypervirulent strains (D_h_). The rate at which individuals are infected is governed by the transmission coefficient (β). Once infected, individuals subsequently become colonized by the pathogen at rate ‘η’. Most of these individuals will remain asymptomatic (determined by parameter ε) until the infection resolves and will re-enter the ‘unexposed’ category. The remaining individuals who go on to experience symptomatic CDI either self-resolve (re-entering the unexposed category), or revert to asymptomatically colonized or they die (according to rate μ). Births are set to perfectly balance deaths to maintain a stable human population, ϕ = μ(D + D_h_). The parameters governing the rates of change and the associated proportions are described in Table 1.

The key mechanisms by which hypervirulent strains differ from normal endemic strains are: 1) the rate of transmission is higher for hypervirulent strains (β_h _> β); 2) the proportion that experience symptomatic disease is higher for hosts infected with hypervirulent strains (ε_h _> ε); and 3) individuals that are already colonized with normal endemic strains can be colonized by hypervirulent strains (α > 0). To ascertain the effects of these three alternative mechanisms, 1000 stochastic introductions of a hypervirulent strain into a community that already harboured normal endemic *C. difficile* at stable equilibrium was simulated. Several epidemiologically relevant metrics were evaluated: the proportion of introductions that elicited an epidemic; the speed at which the newly introduced strain equilibrated; and the new equilibrium prevalence level following successful invasion, across a broad range of the parameters governing the different mechanisms of hypervirulence.

## Results

**Figure 2:**
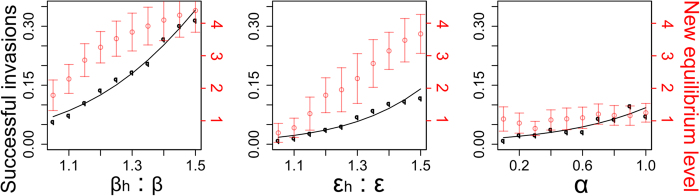
Proportion of hypervirulent *Clostridium difficile* introductions that successfully invade (along with fitted logistic regression curves; black left y-axis) and the new level to which they equilibrate (per 10,000 individuals, red right y-axis). Output from 1000 simulated introductions are displayed for each parameter level under all mechanistic scenarios. Left plot: hypervirulent strains are more infectious (modelled by increasing the transmission coefficient); Middle plot: hypervirulent strains give rise to a higher symptomatic rate; Right plot: hypervirulent strains can outcompete endemic strains in the host’s gut.

Establishment (i.e. successful invasion) of hypervirulent strains was more likely for higher simulated levels of infectiousness (left axis, Fig. 2). For example, an invading strain that is 50% more infectious than endemic strains successfully established in 31.6% of simulations, compared with a strain that is only 20% more infectious which established in 13.8% of simulations. Similarly, hypervirulent strains that elicited a higher symptomatic rate in colonized individuals and that were capable of infecting individuals already colonized by normal endemic strains were more likely to establish. For example, 11.8% of invasions established for hypervirulent strains eliciting a 50% increase in the symptomatic rate relative to typical endemic strains whereas only 3.8% of invasions established with strains eliciting a 20% increase in the symptomatic rate relative to endemic strains. Seven percent of hypervirulent invasions became established when individuals who were colonized with endemic strains were equally susceptible to infection as uncolonized individuals, but only 2.4% of invasions established if endemic-colonized individuals were only one-fifth as susceptible to hypervirulent infection as uncolonized individuals.

Hypervirulence modelled through increased transmission potential, or through increased symptomatic infection rate, also had a positive relationship with the new equilibrium level established by the invading pathogen (right axis, Fig. 2). For example, an increased infectiousness associated with hypervirulence of 20% led to a new equilibrium prevalence of 3.2 symptomatic infections per 10,000 individuals (s.d. 0.5) compared to a 50% increased infectiousness which led to new equilibrium prevalence of 4.4 symptomatic infections per 10,000 individuals (s.d. 0.7). However, the third modelled mechanism of hypervirulence (an ability of hypervirulent strains to displace endemic strains within the host gut) showed no apparent relationship with the resultant new equilibrium prevalence.

**Figure 3:**
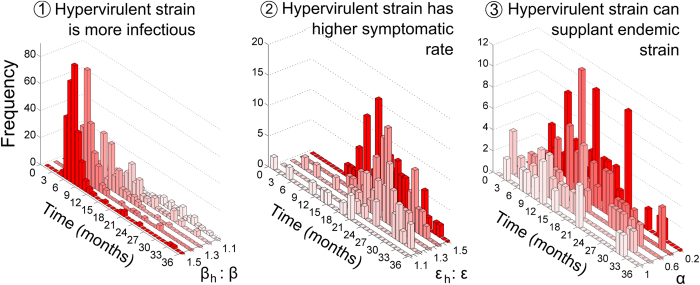
Frequency distribution of time (in months) taken for a newly introduced hypervirulent strain of *Clostridium difficile* to establish new equilibrium under the different mechanisms of hypervirulence. Output from 1000 simulated introductions are displayed for each parameter level under all mechanistic scenarios (note the reversed parameter axes for β and for α which was done to display results more clearly).

The speed of establishment had a positive relationship with the level of hypervirulent strain infectiousness relative to endemic strains (Fig. 3); and a similar relationship was observed between establishment speed and hypervirulent strains eliciting higher symptomatic rates. Again, when hypervirulence was modelled by allowing an ability of hypervirulent strains to displace endemic strains within the host gut, the relationship between speed of establishment and displacement ability was obscured (Fig. 3).

**Figure 4:**
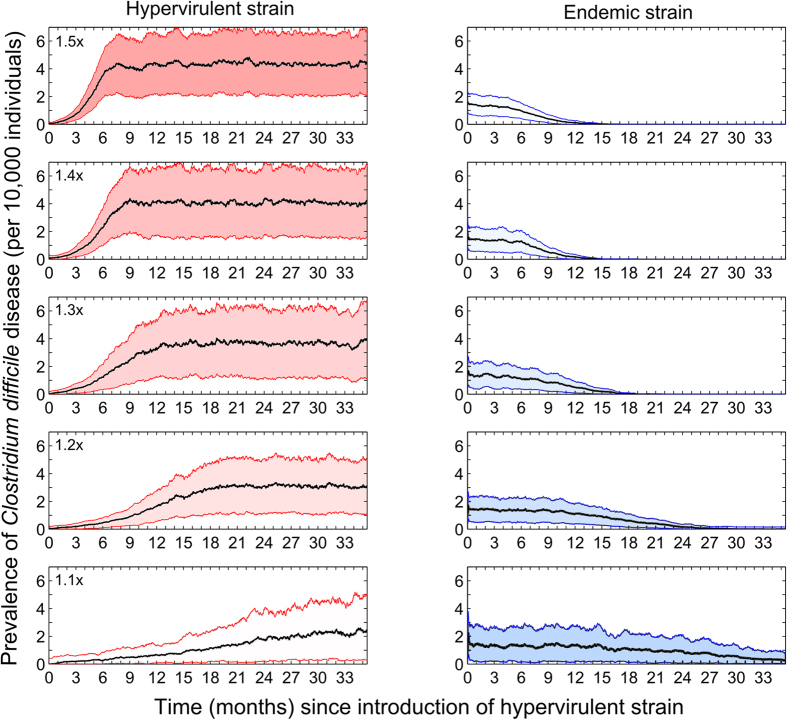
Successfully invading hypervirulent strains of *Clostridium difficile* (left column, red) competitively exclude pre-existing endemic strains (right column, blue). The fold increase in infectiousness relative to endemic strains is shown in the top left of the hypervirulent plots. A one-month moving average of the successful hypervirulent strain invasions from 1000 simulated introductions are displayed (with shaded 95% confidence intervals).

Results demonstrate that regardless of the modelled mechanism of hypervirulence, the successful invasion of the introduced hypervirulent strain resulted in the exclusion of the extant endemic strain (Fig. 4).

## Discussion

Through stochastic simulation, the invasion of a hypervirulent strain of *Clostridium difficile* into a human community already harbouring an endemic strain was explored. Several mathematical models of *C. difficile* transmission have been reported^22^,^23^, most having been published in the last five years^24^,^25^,^26^,^27^,^28^,^29^. The rationale behind all these previous models was to strategize the control of infection in a hospital setting. However, *C. difficile* is increasingly recognised as a pathogen of the global community, rather than just the subset of the community housed within healthcare facilities. Additionally, recent studies have suggested that the community is a major source, if not the primary source, of infections experienced by the high-risk groups within healthcare settings^30^,^31^. To the best of our knowledge, the current study constitutes the first epidemiological model of *C. difficile* transmission within the wider community as well as the first comparative analysis of alternative mechanisms of hypervirulence.

Precise causes for the difference in virulence between hypervirulent stains and endemic strains remain unknown despite the fact that these newer ‘atypical’ strains now constitute the majority of infections in the community setting^32^. Consequently, the effects of three different mechanisms of heightened virulence were tested: increased infectiousness of the pathogen, an increased rate of symptomatic disease following colonization, and the ability of hypervirulent strains to displace endemic strains from a colonized gut. Intuitively, the parameters governing these different mechanisms all had positive relationships with the probability of an invading strain establishing in the community. However, comparing the influence of these parameters on the rate of invasion and the resultant equilibrium prevalence yielded strikingly different epidemiological patterns.

In line with classic epidemiological understanding^33^, the rate at which an introduced pathogen spreads among a susceptible population is highly dependent on the transmission coefficient, which was modelled by increasing the infectiousness of a hypervirulent strain. Simulations showed that more infectious strains were more likely to establish, spread more rapidly, and equilibrated to a higher prevalence within the community. The likelihood of successful invasion and the new steady state prevalence were both less dramatically influenced by increasing the colonized proportion that went on to experience clinical disease. When individuals colonized with endemic strains were susceptible to colonization with hypervirulent strains (the third modelled mechanism of hypervirulence) a much weaker relationship was found with likelihood of establishment, and no clear relationship was seen with the resulting equilibrium prevalence. This is because the spread of the newly introduced strain is essentially independent of the resident strain endemicity when a resident strain-colonized gut is colonized just as readily as an uncolonized gut. Consequences of this finding for the strategy to reduce hypervirulent spread through artificial infection with non-toxigenic strains require exploration.

Clinical reports during the past 15 years have described significantly increased rates of disease corresponding with a pronounced and rapid shift in *C. difficile* strain dominance. PCR-ribotyping of isolates from a Montreal area hospital demonstrated that NAP1/ribotype 027 was absent in 2000 and 2001 but represented more than 75% of all isolates corresponding with an outbreak in 2003–2004^34^. Increased disease prevalence has corresponded with the dominance of ribotype 027 in numerous countries across the world including in England where it peaked in 2007–2008^35^, in Europe^36^ and North America^37^.

Tying this epidemiological picture in with the results of the current analysis, it appears that an ability of hypervirulent strains to displace endemic strains from the already-colonized host gut is the least likely mechanism facilitating dominance of ribotype 027. Despite testing a broad range of parameter values, from complete colonization resistance to susceptibility equivalent to an uncolonized individual, the newly introduced strain failed to reproduce the heightened prevalence level associated with emerging hypervirulent strains. This finding does not negate the possibility that hypervirulent strains are more competitive within-host than more typical strains; but it does suggests that this mechanism is not key to the successful invasion and clonal dominance of hypervirulent strains such as ribotype 027. Importantly, the current study demonstrated that direct competition between strains (inside the host’s gut) is not a prerequisite for the sudden switching in prevailing strains; simulations of all alternative hypervirulence mechanisms clearly illustrated that previously dominant strains are not simply added to following new strain invasion, but are excluded through indirect (exploitative) competition.

Transmission dynamics of the remaining alternative hypervirulence mechanisms (increased infectiousness and increased symptomatic disease) are much more similar and, therefore, will be much more difficult to disentangle. It is likely that distinguishing between the remaining alternatives will not be possible from comparisons of simulation output with longitudinal, ribotyped infection data, and will necessitate a much clearer clinical picture of *C. difficile* infection. When these data become available in the future and/or there is increasing evidence derived through alternative means that favours a particular mechanism of hypervirulence, the current model formulation offers an important epidemiological tool for contributing towards infection control strategy. These developments will in turn allow for better refinement of the model to account for the interaction between host (as well as bacterial) factors involved in pathogenesis.

There are a number of limitations to the current study that warrant discussion. Despite burgeoning interest in this pathogen of global health significance, basic metrics of the infection process, such as latent periods, are scant in the literature^38^. Due to limited information on the life history of *C. difficile* infection, parameterisation of the current model has depended on numbers amassed from multiple studies across multiple epidemiological settings. This is a common issue with biologically realistic simulation modelling^39^. While a substantial effort was made in preferentially selecting recent studies that better reflected the pathogen’s modern epidemiology (published within the past 5 years) as sources of parameter estimates, this was not always possible. Another important limitation is that it has been assumed that the alternative hypervirulence mechanisms operate in a mutually exclusive manner when, in reality, several mechanisms might interact synergistically. Perhaps the most important limitation is the absence of longitudinal ribotype data for a newly invading hypervirulent strain with which to fit our simulation model. The current study using the most up-to-date clinical and microbiological information demonstrates that a complete switch in the dominant ribotype can take place in as little as 6 months. This highlights not only the frequency at which ribotype data would require collection to capture invasion dynamics but also the necessity for an extremely rapid, active surveillance response following initial hypervirulent detection.

Over the past 15 years, morbidity and mortality resulting from *C. difficile* has steadily increased worldwide as a function of the emergence of hypervirulent strains (most notably, ribotype 027). There is contention surrounding all currently proposed mechanisms distinguishing hypervirulent strains from more typical (less virulent) predecessor strains; how this pathogen has become the leading cause of infectious nosocomial diarrhoea remains unknown. In addition to providing new evidence that clearly favours certain hypervirulence mechanisms over others, the current analysis constitutes the first epidemiological model to explore the dynamics of *C. difficile* outside of a healthcare setting by simulating pathogen spread within the wider human community – an aspect that is widely regarded to be critical to the pathogen’s modern epidemiology. Methods described in this foundational study provide an important contribution to future outbreak analysis of this disease of increasing global relevance.

## Additional Information

**How to cite this article**: Yakob, L. *et al.* Mechanisms of hypervirulent *Clostridium difficile* ribotype 027 displacement of endemic strains: an epidemiological model. *Sci. Rep.* **5**, 12666; doi: 10.1038/srep12666 (2015).

## Figures and Tables

**Table 1:** Epidemiological model symbology and parameterisation.

**Symbol**	**Definition**	**Value**	**Ref.**
*β*_*h*_ *: β*	Transmission coefficient (hypervirulent:endemic strain) **MECHANISM 1**	1–1.5	Full range tested in simulations
*ε*_*h*_ : ε	Develop symptoms (proportion) (hypervirulent:endemic strain); **MECHANISM 2**	1–1.5	
*α*	Hypervirulent strain’s ability to supplant colonized endemic; **MECHANISM 3**	0–1	
1-*ε*	Colonization self-clearance (proportion)	0.8	40
*η*	Develop into asymptomatic but Infectious state (day^−1^)	0.2	41
*θ*	Develop symptomatic CDI (day^−1^)	0.2	40
*ζ*	CDI self-resolve (proportion)	0.33	42
*τ*	CDI self-resolve rate (day^−1^)	0.5	43
*ρ*	CDI treatment (day^−1^)	0.1	44
*σ*	Treatment failure (proportion)	0.2	45
*μ*	Mortality rate (day^−1^)	0.0012	40
